# Editorial: Cellular and molecular pathologies of Alzheimer’s disease: understanding the link between different etiological factors for therapeutic exploitation

**DOI:** 10.3389/fcell.2026.1813923

**Published:** 2026-03-12

**Authors:** Mohammad Golam Sabbir

**Affiliations:** 1 Nova Southeastern University, Fort Lauderdale, FL, United States; 2 Capillus, Miami, FL, United States

**Keywords:** Alzheiemer’s disease, calcium/calmodulin-dependent kinase kinase 2, epigenetic modulation, fangchinoline, m5C RNA methylation, O-GlcNAcylation, proteostais, TREM2 (triggering receptor expressed on myeloid cells 2)

## Introduction

Alzheimer’s disease (AD) remains a clinicopathological syndrome potentially emerging from the convergence of a multitude of seemingly unrelated but possibly interlinked or interdependent pathogenic pathways (Korczyn and Grinberg, 2024). Since Alois Alzheimer first described the disorder in 1906 (Hippius and Neundörfer, 2003), numerous hypotheses have been advanced to explain its pathogenesis - including the amyloid and tau hypotheses, dysregulated calcium signaling, brain iron overload, neuroinflammation, and genetic influences that interact with age-related transcriptional dysregulation as well as epigenetic and epitranscriptomic alterations. No single hypothesis fully accounts for the heterogeneity or progression of AD. Moreover, the brain’s cellular complexity adds further layers: neurons (sites of synaptic failure and degeneration), microglia and astrocytes (injury responses, phagocytosis, and synaptic remodeling), oligodendrocytes (myelin integrity), and the neurovascular unit - comprising endothelial cells, pericytes, and associated glia (barrier function and iron transport) - all participate. The dynamic interplay among these cell types and signaling networks both complicates our understanding of AD and highlights the need for integrative, mechanism-informed therapeutic strategies. Therefore, the goal of this Research Topic was to move beyond single-pathway silos and illuminate mechanistic links among different etiologies - so that future interventions can be rationally combined, timed, and measured. Seven contributions in this collection collectively map a set of convergent circuits spanning nuclear tau–chromatin remodeling, calcium–iron dyshomeostasis, glial state transitions (Triggering Receptor Expressed on Myeloid cells 2: TREM2), autophagy and redox biology, and infection-driven RNA methylation.

## Tau as a chromatin remodeler shifting transcriptional landscape during AD pathogensis

Siano and colleagues provided evidence that soluble, nuclear microtubule associated protein tau (MAPT/tau) associated with reshaping the neuronal transcriptome in a manner that most strongly resembles intermediate (proAD) stages of human AD, not the earliest or latest phases. Mechanistically, tau overabundance associates with a reduction in heterochromatin markers - heterochromatin protein one alpha (HP1α) and histone H3 lysine nine trimethylation (H3K9me3) – both *in vitro* cell-based systems and in human temporal cortex, and the differentially expressed genes align with histone acetylation changes observed in AD brains. These findings support a model in which nuclear tau drives epigenetic modulation and transcriptional reprogramming prior to irreversible degeneration in AD.

## Calcium meets iron: calcium/calmodulin dependent protein kinase kinase 2 (CAMKK2), transferrin pathways, and oxidative stress

Two retrospective postmortem human tissue-based studies by Sabbir et al. indicated convergence on a Ca^2+^-iron intersection point regulated by CAMKK2. In hippocampus, Sabbir et al. reported reduced CAMKK2 and transferrin (TF) across AD, frontotemporal degeneration (FTD), and parkinson’s disease (PD), with TF receptor (TFRC) reduction specific to late-onset AD; tau is increased with aggregate-like smears specifically in AD. Strikingly, CAMKK2/TF correlations present in controls are lost in AD, suggesting disease-specific decoupling of calcium and iron trafficking.

Complementing this, a large postmortem temporal cortex cohort shows decreased CAMKK2, TF, and TFRC in AD (and TFRC in PD as well), alongside increased iron content correlating with TF/TFRC levels - implicating disrupted receptor-mediated iron handling in neurotoxicity (Sabbir, 2024). Together these studies position CAMKK2 as a mechanistic hub linking Ca^2+^ signaling to iron homeostasis, with regional and stage specificity: TFRC loss emerged late in hippocampus but was evident earlier in cortex. Translationally, they argue for biomarkers that pair quantitative susceptibility mapping (QSM) Magnetic resonance imaging for measuring brain iron content (Madden and Merenstein, 2023; Chen et al., 2025) with a plasma or cerebrospinal fluid (CSF) TF/TFRC and AD biomarker panels (amyloid-β 42/40 ratio, phosphorylated tau at threonine 181, glial fibrillary acidic protein, neurofilament light chain, phosphorylated tau at threonine 217 and apolipoprotein E ε4 allele) (González-Escalante et al., 2025), and for therapies that restore CAMKK2 axis activity or stabilize TF/TFRC trafficking.

## Microglia at the crossroads: TREM2, tau, and timing

Huang et al. reviewed the dual, stage-dependent roles of TREM2 in tauopathy. TREM2 signaling can suppress glycogen synthase kinase three beta/cyclin-dependent kinase 5 (GSK3β/CDK5) activity and reduce tau phosphorylation; yet chronic or mis-timed activation may exacerbate amyloid-β (Aβ)-associated tau seeding and spread. Soluble TREM2 (CSF) rises early during AD pathogenesis and correlates with t-tau/p-tau, and genetic variants of TREM2, notably R47H (arginine-to-histidine) modulate microglial phenotypes. These dynamics reinforce a precision-timing concept: TREM2 agonism may be beneficial when it promotes disease-associated microglia stage 2 (DAM2) transitions, but harmful if it sustains microgliosis without clearing Aβ in seeding-permissive contexts.

## Proteostasis and redox: autophagy and antioxidant defenses

Yi et al. demonstrate that fangchinoline (a natural bisbenzylisoquinoline) promotes autophagy–lysosome degradation of beta-secretase 1 **(**BACE1), reduces amyloidogenic amyloid beta precursor protein (APP) processing, and improves cognition in an Aβ1–42 mouse model while enhancing antioxidant programs (nuclear factor erythroid 2–related factor 2/heme oxygenase-1/superoxide dismutase 1)and reducing hydrogen peroxide (H_2_O_2_) and inducible nitric oxide synthase (iNOS). While a single plant derived alkaloid cannot address AD’s full complexity, these data highlight autophagy induction as a practical lever that simultaneously lowers Aβ drive and attenuates oxidative stress, thereby relieving pressure on pathways that may fuel AD pathogenesis.

## Glial interface: what O-GlcNAc augmentation did - and did not - do

The O-linked-β-N-acetylglucosamine (O-GlcNAc) post-translational modification - addition of a single *N*-acetylglucosamine (GlcNAc) to serine/threonine residues - has been proposed to modulate the amyloidogenic processing of APP, motivating pharmacological strategies that enhance O-GlcNAcylation. Building on this rationale, Garcia et al. tested the selective O-GlcNAcase (OGA) inhibitor thiamet-G to increase O-GlcNAcylation and potentially alleviate progressive AD pathology in female transgenic TgF344-AD rats. The results were mixed: plaque burden did not change, microglial reactivity did not increase, and noradrenergic axon density was not rescued. By contrast, astrocytes adjacent to plaques showed greater morphological complexity (consistent with a more encapsulating “glial border”), and dystrophic axon morphology was reduced. Overall, these data suggest an astrocyte-proximal benefit coexisting with neutral effects on plaques and microglia, reinforcing the need for cell- and compartment-specific readouts when evaluating metabolic/proteostasis interventions.

## Infection and the epitranscriptome: m^5^C methylation as an integrator

Teng et al. review how pathogen exposure (viruses, bacteria, parasites) may shape AD risk and progression not only through inflammation and barrier injury, but also by modulating RNA 5-methylcytosine (m^5^C) in host cells. The writers/readers/erasers (e.g., NSUN, ALYREF, TET family proteins) influence RNA stability and immune programs; alterations in m^5^C have been linked to pathogen replication, host antiviral signaling, and, increasingly, to neuropathology and cognition. This positions epitranscriptomic drift - potentially infection-triggered- as a plausible upstream driver that intersects with the nuclear tau–chromatin axis and with microglial immune state via transcript control.

Collectively, the contributions in this Research Topic argue that AD is best understood as a network disorder in which neuronal, glial, vascular, and immune programs interact across time through a set of convergent axes: nuclear tau–chromatin remodeling that reprograms transcription at intermediate stages; a Ca^2+^-CAMKK2-transferrin signaling hub that links calcium signaling to brain iron handling; microglial state transitions gated by TREM2 that can contain or propagate tau; proteostatic control via autophagy that lowers amyloidogenic drive and oxidative stress; and infection-linked m^5^C epitranscriptomic drift that may recalibrate innate immune tone and intersect with amyloid/tau biology. Together, these insights motivate an integrated, stage-aware roadmap for therapy development.

## References

[B1] Chen L. Soldan A. Faria A. Albert M. van Zijl P. C. M. Li X. (2025). Susceptibility mri helps predict mild cognitive impairment onset and cognitive decline in cognitively unimpaired older adults. Radiology 316 (3), e250513. 10.1148/radiol.250513 40923890 PMC12501624

[B2] González-Escalante, A. Milà-Alomà M. Brum W. S. Ashton N. J. Ortiz-Romero P. Shekari M. (2025). A plasma biomarker panel for detecting early amyloid-β accumulation and its changes in middle-aged cognitively unimpaired individuals at risk for alzheimer’s disease. eBioMedicine 116, 105741. 10.1016/j.ebiom.2025.105741 40414160 PMC12159936

[B3] Hippius H. Neundörfer G. (2003). The discovery of Alzheimer'S disease. Dialogues Clin. Neurosci. 5 (1), 101–108. 10.31887/DCNS.2003.5.1/hhippius 22034141 PMC3181715

[B4] Korczyn A. D. Grinberg L. T. (2024). Is alzheimer disease a disease? Nat. Rev. Neurol. 20 (4), 245–251. 10.1038/s41582-024-00940-4 38424454 PMC12077607

[B5] Madden D. J. Merenstein J. L. (2023). Quantitative susceptibility mapping of brain iron in healthy aging and cognition. Neuroimage 282, 120401. 10.1016/j.neuroimage.2023.120401 37802405 PMC10797559

[B6] Sabbir M. G. (2024). Loss of calcium/calmodulin-dependent protein kinase kinase 2, transferrin, and transferrin receptor proteins in the temporal cortex of alzheimer's patients postmortem is associated with abnormal iron homeostasis: implications for patient survival. Front. Cell Dev. Biol. 12, 1469751. 10.3389/fcell.2024.1469751 39669708 PMC11634808

